# Congenital Porto-Azygous Shunt (Abernethy Malformation Type II) in an Elderly Patient: A Too-Often-Forgotten Occult Abnormality

**DOI:** 10.7759/cureus.24460

**Published:** 2022-04-25

**Authors:** Steven Tessier, Firas Ido, Thomas Zanders, Santo Longo, Sudip Nanda

**Affiliations:** 1 Department of Pathology, St. Luke's University Health Network, Bethlehem, USA; 2 Department of Pulmonary and Critical Care Medicine, St. Luke's University Health Network, Bethlehem, USA; 3 Department of Cardiology, St. Luke's University Health Network, Bethlehem, USA

**Keywords:** elderly, hyperammonemia, portosystemic shunt, malformation, abernethy

## Abstract

Congenital extrahepatic portosystemic shunts (CEPS) cause portal blood to circumvent the liver and its metabolism, allowing normally detoxified ammonia to accumulate in the systemic circulation. Hyperammonemia in the elderly often manifests clinically as toxic encephalopathy. We present a case of recurrent altered mental status in a 70-year-old patient that eluded diagnosis over several years. Hyperammonemia was the sole abnormality detected upon a thorough liver function evaluation prompted by the patient’s history of remote liver disease. Enhanced computed tomography revealed an extrahepatic porto-azygous shunt arising from a hypoplastic portal vein. This case illustrates that, albeit rare, CEPS may express themselves for the first time in the elderly, a patient population that is frequently afflicted by many more common causes of altered mental status. CEPS should be considered in the differential diagnosis of inexplicable hyperammonemia in this age group.

## Introduction

Over 200 years ago, the English surgeon John Abernethy discovered an “uncommon formation in the viscera of the human body”, indeed a congenital portocaval shunt, while performing a postmortem examination on a 10-month-old female infant [1]. This case was the first in historical literature that inspired the eponym “Abernethy malformation”. Abernethy malformations are also known as congenital extrahepatic portosystemic shunts (CEPS). CEPS arises by abnormal regression of paired vitelline and umbilical vein segments during embryogenesis, forming veno-venous communications between the portal and systemic circulations [2]. The rarity of CEPS has precluded firm consensus on their incidence. However, rough estimates inferred from neonatal screenings for hypergalactosemia suggest CEPS occurs one in every 30,000 births (CEPS allows galactose to bypass the liver) [3,4]. The Morgan and Superina criteria are commonly used to classify CEPS into two major subtypes [5]. Type I CEPS are distinguished by an absence of the portal vein (end) and complete hepatic bypass (side), forming an end-to-side shunt. Type II CEPS are side-to-side shunts as some liver perfusion is attained by the presence of a small-caliber portal vein. This results in sub-normal hepatic perfusion. In both types of shunts, the majority of portal blood bypasses hepatic metabolism by directly entering the systemic circulation. A notable consequence is the accumulation of ammonia in the blood, which can result in hyperammonemic encephalopathy. Other associations with CEPS include pulmonary hypertension, hepatocellular carcinoma, liver adenomas, and structural malformations such as biliary atresia and polysplenia [6]. Symptoms caused by type II CEPS typically arise in childhood and early adulthood. Very few cases have been reported in the elderly [7-10]. Here, we present a case of hyperammonemic encephalopathy in a 70-year-old patient who was found to have an extrahepatic porto-azygous shunt stemming from a hypoplastic portal vein (CEPS type II).

## Case presentation

**Figure 1:**
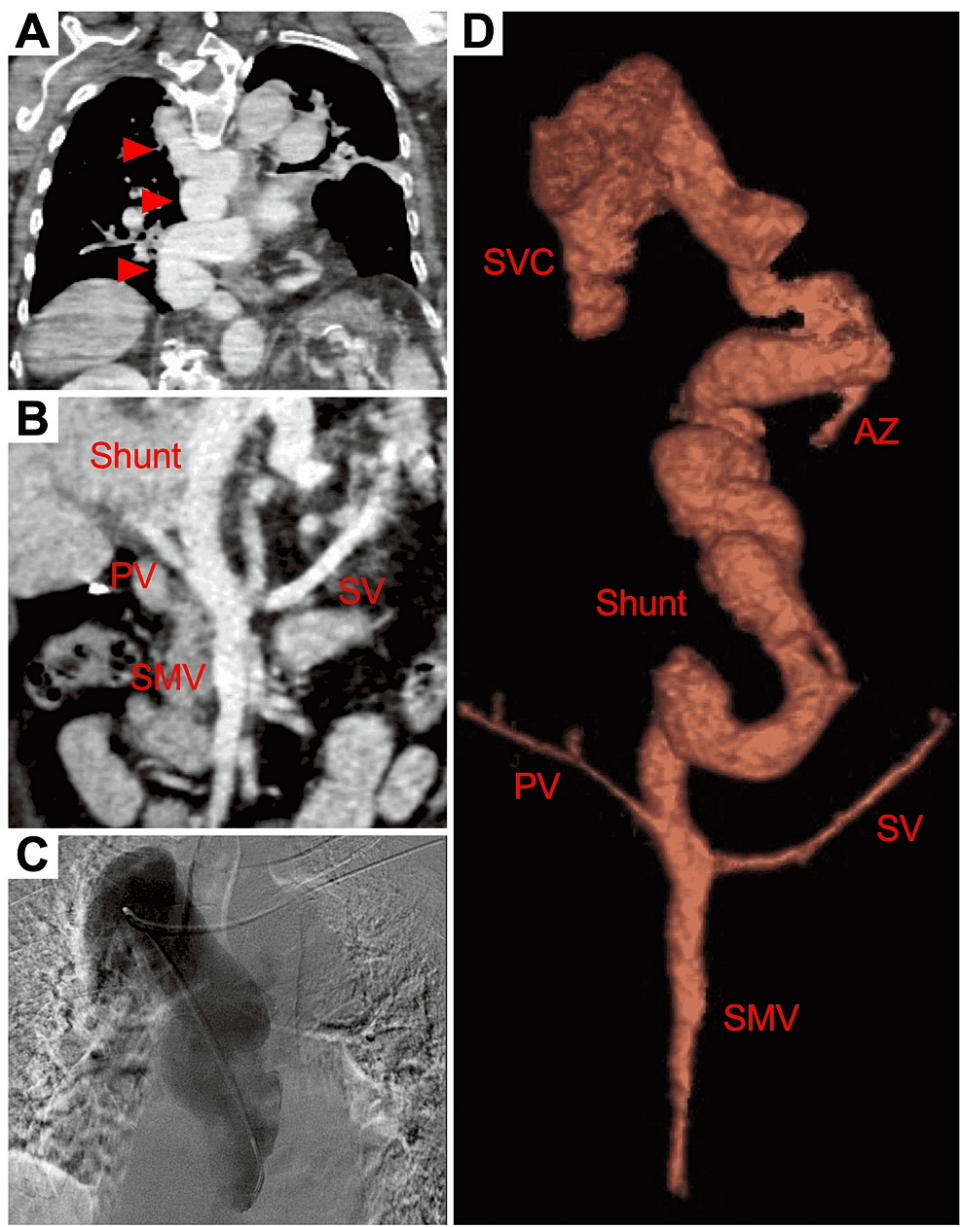
Porto-azygous shunt. A: Chest CT of the massive shunt (red arrowheads); B: CT below the diaphragm showing the superior mesenteric and splenic vein merging together to form the portal vein, which gives off the shunt; C: Angiogram of the intrathoracic shunt; D: 3D reconstruction of the shunt as it merges with the azygous vein above the diaphragm at the level of T7; SMV: Superior mesenteric vein; SV: Splenic vein; PV: Portal vein; AZ: Azygous vein; SVC: Superior vena cava.

The patient is a wheelchair-bound 70-year-old female with a past medical history of insulin-dependent diabetes mellitus, chronic diastolic congestive heart failure, obstructive pulmonary disease and hypercarbic respiratory failure with oxygen dependency, morbid obesity status post-Roux-en-Y surgery, and recurrent admissions for altered mental status. She was found at home by a visiting nurse obtunded, only occasionally opening her eyes, and minimally responsive to noxious stimuli. Upon urgent transportation to the hospital, Emergency Medical Services noted room air SpO_2_ of 85%, left facial droop, right hemiplegia, and sporadic limb myoclonus. A nasal cannula and a non-rebreather mask improved her saturation to the high 90's. She was hypertensive with a blood pressure of 187/92 mmHg. Her heart rate was 93 beats per minute, her respiratory rate was 18 bpm, and she was afebrile. Fingerstick glucose was 150-160 mmol/L. On arrival at the emergency department, the patient was intubated for airway protection. At this time, her eyes rolled into a fixed position with gaze deviation and there was posturing of the upper extremities. Concern was present for stroke and/or seizure. Immediate computed tomography (CT) of the head and CT angiogram were negative for acute stroke. Physical exam after holding sedation upon arrival to the intensive care unit revealed unresponsiveness to sternal rub, minimal withdrawal to painful stimuli, an upward and leftward gaze deviation, and intact gag and cough reflexes. Continuous video electroencephalogram (EEG) demonstrated periodic limb movements that coincided with non-epileptiform triphasic wave patterns consistent with diffuse cerebral dysfunction. Laboratory studies including a liver function panel were normal except for an elevated ammonia level of 272 µmol/L. Autoimmune and viral hepatitis serology were negative. Ammonia levels were corrected to 28 µmol/L with lactulose 20 g three times daily. Two days after admission, the patient remained intubated but became alert to voice with the ability to follow commands. A follow-up EEG revealed no electrographic seizures and the triphasic waves had markedly reduced. Liver ultrasound and hepatic core needle biopsy demonstrated normal echotexture and mild steatosis (10%) without cirrhosis, respectively. Chest and abdomen contrast-enhanced CT revealed an extrahepatic porto-azygous vascular shunt arising from a persistent hypoplastic portal vein (Figure 1). This shunt was tortuous and massive, measuring 3 cm in the greatest dimension. The azygous vein was severely distended, 7 mm in size. It was the consensus that this anatomic anomaly was a type II CEPS. Consideration of shunt reduction by surgical intervention would be determined on close follow-up of the patient by referral to a liver specialty center.

## Discussion

This is a case of an unusual and rare presentation of hyperammonemia. CEPS typically involve direct caval anastomoses. This case, however, demonstrates an atypical variant with a direct azygous connection. Despite having a congenital malformation, our 70-year-old patient remained asymptomatic for most of her life. The tardive unveiling of the shunt likely involved multiple factors, most notable of which is the patient’s history of Roux-en-Y surgery.

Five years before a diagnosis of CEPS was made, the patient underwent Roux-en-Y as a treatment for morbid obesity. The Roux-en-Y procedure connects a small stomach pouch with the small intestine to bypass the duodenum and the upper portion of the jejunum. The result is a restriction of food intake and malabsorption of nutrients together reducing calories and effectively promoting weight loss. Such a reduction in nutrition encourages catabolism by protein breakdown in extra-hepatic tissues, followed by deamination and transamination of the freed amino acids [11]. Furthermore, Roux-en-Y may alter the gut microbiome that participates in the metabolism of ammonia [11]. It is possible that factors related to the patient’s Roux-en-Y procedure increased the burden of nitrogenous waste and thus contributed to the late-onset manifestation of shunt-associated symptoms. Other potentially contributing factors include the patient’s congestive heart failure and obesity.

This case underscores the importance of expanding the differential diagnosis to include CEPS when a patient presents with hyperammonemia in the absence of overt liver disease. While less likely, this view should be upheld even in the elderly. Common causes of altered mental status in the elderly can be grouped into 4 categories: systemic disease, primary central nervous system (CNS) disease, drugs, and environmental/iatrogenic [12]. Acute and chronic causes are listed in Table 1 [12,13]. 

**Table 1:** Causes of Altered Mental Status in the Elderly. Adapted from Wilber and Ondrejka [12] and Inouye [13].

Systemic Disease and Acute Illness	Primary Diseases of the CNS	Drugs	Environmental and Iatrogenic Causes
Infection/sepsis	Dementia	Polypharmacy	Prolonged ED/Hospital stay
Shock	Meningitis	Alcohol/sedative withdrawal	Sleep deprivation
Hypoxia	Encephalitis	Recreational drug/alcohol use	Physical restraints
Hypercarbia	Seizures/postictal state	Anticholinergics	Indwelling urinary catheter
Dehydration	Stroke	Sedative-hypnotics	Surgery or procedures
Electrolyte abnormalities	Subdural hemorrhage	Opioids	
Hypo/hyperglycemia	Epidural hemorrhage		
Hypo/hyperthermia	Intracranial hemorrhage		
Trauma			
Myocardial infarction			
Malnutrition/starvation			
Anemia			
Low serum albumin			

In addition, the causes of hyperammonemia in adults are listed in Table 2 [14]. Included are common causes such as urinary tract infections, cirrhosis, and medications, as well as rare causes such as urea cycle disorders. Differentiating these entities can be performed objectively through laboratory and imaging studies. Urinary tract infections with urease-positive organisms (Proteus, *E. coli*, Klebsiella) can be detected through urinalysis and urine culture. The presence of cirrhosis is typically marked by hepatic changes on imaging (ultrasound or CT), which demonstrates a nodular hepatic contour, and laboratory findings such as elevated transaminases and prothrombin time (or INR). The serum level of medications such as valproic acid can be measured to determine whether the level is above therapeutic range. Urea cycle disorders typically present in neonates; however, these disorders can be detected later in life in patients with partial enzyme deficiencies. The diagnosis can be made by measurement of amino acid levels in the blood, in particular citrulline and argininosuccinic acid.

**Table 2:** Differential Diagnoses for Hyperammonemia. Adapted from Clay and Hainline [14].

Increased Production of Ammonia	Decreased Elimination of Ammonia
Gastric bypass	Portosystemic shunt
Infection i.e. urease producing bacteria	Liver failure (including drug-induced i.e. acetaminophen)
Increased protein load	Drugs i.e. valproic acid, carbamazepine, salicylates, glycine, sulfadiazine, pyrimethamine
Intense exercise	Urea cycle disorders/inborn errors of metabolism
Seizures	Idiopathic hyperammonemia
Trauma/burns	
Steroids	
Chemotherapy	
Malnutrition/starvation	
Gastrointestinal hemorrhage	
Total parenteral nutrition	
Cancer	

Once diagnosed, symptomatic CEPS requires intervention. Management recommendations include endovascular or surgical closure of type II CEPS and liver transplantation in patients with type I CEPS. These recommendations are based on the aforementioned Morgan and Superina classification system. Of note, emerging evidence suggests that hypoplastic albeit functional portal systems do exist in type I shunts and can be visualized by balloon occlusion venography [15,16]. Thus, shunt closure of type 1 CEPS without the requirement of liver transplantation may be possible [16]. Rare complications of shunt closure include deterioration of pulmonary hypertension, acute portal hypertension, and thrombosis; close monitoring after the operation is recommended [17]. Complication risk may be mitigated preoperatively by revealing shunt anatomy via venography and simulating shunt closure hemodynamics by balloon occlusion [16]. A standardized treatment strategy is not yet established given the complexity and rarity of this congenital malformation.

## Conclusions

Altered mental status in the elderly patient is a common everyday challenge to caregivers. Its differential diagnosis includes liver disease, metabolic and drug toxicity, primary neurologic abnormalities, and environmental and iatrogenic causes. Hyperammonemic encephalopathy in the absence of clinically or chemically detectable liver dysfunction requires a high index of suspicion for an occult portal vein circulation abnormality. As reported herein and by others, CEPS can remain undetected late into adulthood. Although extremely rare in the elderly patient, our case illustrates that CEPS should be included in the differential diagnosis of hyperammonemia in this age group.

## References

[1] Abernethy J (1797). Account of two instances of uncommon formation in the viscera of the human body: from the philosophical transactions of the Royal Society of London. Med Facts Obs.

[2] Ghandour A, Partovi S, Karuppasamy K, Rajiah P (2016). Congenital anomalies of the IVC-embryological perspective and clinical relevance. Cardiovasc Diagn Ther.

[3] Ono H, Mawatari H, Mizoguchi N, Eguchi T, Sakura N (1998). Clinical features and outcome of eight infants with intrahepatic porto-venous shunts detected in neonatal screening for galactosaemia. Acta Paediatr.

[4] Gitzelmann R, Forster I, Willi UV (1997). Hypergalactosaemia in a newborn: self-limiting intrahepatic portosystemic venous shunt. Eur J Pediatr.

[5] Morgan G, Superina R (1994). Congenital absence of the portal vein: two cases and a proposed classification system for portasystemic vascular anomalies. J Pediatr Surg.

[6] Baiges A, Turon F, Simón-Talero M (2020). Congenital extrahepatic portosystemic shunts (abernethy malformation): an international observational study. Hepatology.

[7] Gómez Contreras R, Talens Ferrando A, Bernal Sprekelsen JC, Landete Molina FJ, Zaragoza Fernández C (2019). Appreciation of the treatment in adult patients with congenital portosystemic connections in relation with their symptoms. Rev Esp Enferm Dig.

[8] Konstas AA, Digumarthy SR, Avery LL, Wallace KL, Lisovsky M, Misdraji J, Hahn PF (2011). Congenital portosystemic shunts: imaging findings and clinical presentations in 11 patients. Eur J Radiol.

[9] Kiriyama M, Takashima S, Sahara H (1996). Case report: portal-systemic encephalopathy due to a congenital extrahepatic portosystemic shunt. J Gastroenterol Hepatol.

[10] Merola E, Cao M, La Starza S, Delle Fave MM, Tavanti F, Sergi D, Marignani M (2016). Portosystemic encephalopathy in an 86-year-old patient : a clinical challenge. Acta Gastroenterol Belg.

[11] Fenves AZ, Shchelochkov OA, Mehta A (2015). Hyperammonemic syndrome after Roux-en-Y gastric bypass. Obesity (Silver Spring).

[12] Wilber ST, Ondrejka JE (2016). Altered mental status and delirium. Emerg Med Clin North Am.

[13] Inouye SK (2006). Delirium in older persons. N Engl J Med.

[14] Clay AS, Hainline BE (2007). Hyperammonemia in the ICU. Chest.

[15] Kanazawa H, Nosaka S, Miyazaki O (2015). The classification based on intrahepatic portal system for congenital portosystemic shunts. J Pediatr Surg.

[16] Rajeswaran S, Johnston A, Green J (2020). Abernethy malformations: evaluation and management of congenital portosystemic shunts. J Vasc Interv Radiol.

[17] Ohno T, Muneuchi J, Ihara K, Yuge T, Kanaya Y, Yamaki S, Hara T (2008). Pulmonary hypertension in patients with congenital portosystemic venous shunt: a previously unrecognized association. Pediatrics.

